# The implications for potential marginal land resources of cassava across worldwide under climate change challenges

**DOI:** 10.1038/s41598-023-42132-y

**Published:** 2023-09-13

**Authors:** Yongping Li, Fangyu Ding, Mengmeng Hao, Shuai Chen, Dong Jiang, Peiwei Fan, Yushu Qian, Jun Zhuo, Jiajie Wu

**Affiliations:** 1 Faculty of Land Resource Engineering, Kunming University of Science and Technology, Kunming, 650093 China; 2 Yunnan Institute of Land Resources Planning and Design, Kunming, 650216 China; 3 State Key Laboratory of Resources and Environmental Information System, Institute of Geographic Sciences and Natural Resources Research, Chinese Academy of Sciences, 11A Datun Road, Chaoyang District, Beijing, 100101 China; 4 College of Resources and Environment, University of Chinese Academy of Sciences, Beijing, 100049 China

**Keywords:** Environmental sciences, Renewable energy, Climate-change ecology

## Abstract

The demand for energy plants is foreseen to grow as worldwide energy and climate policies promote the use of bioenergy for climate change mitigation. To avoid competing with food production, it’s critical to assess future changes in marginal land availability for energy plant development. Using a machine learning method, boosted regression tree, this study modeled potential marginal land resources suitable for cassava under current and different climate change scenarios, based on cassava occurrence records and environmental covariates. The findings revealed that, currently, over 80% of the 1357.24 Mha of available marginal land for cassava cultivation is distributed in Africa and South America. Under three climate change scenarios, by 2030, worldwide suitable marginal land resources were predicted to grow by 39.71Mha, 66.21 Mha, and 39.31Mha for the RCP4.5, RCP6.0, and RCP8.5 scenarios, respectively; by 2050, the potential marginal land suitable for cassava will increase by 38.98Mha, 83.02 Mha, and 55.43Mha, respectively; by 2080, the global marginal land resources were estimated to rise by 40.82 Mha, 99.74 Mha, and 21.87 Mha from now, respectively. Our results highlight the impacts of climate change on potential marginal land resources of cassava across worldwide, which provide the basis for assessing bioenergy potential in the future.

## Introduction

The share of renewable energy resources has been largely increasing in the total primary energy supply^1^. One particular resource is bioenergy, which accounts for roughly one-tenth of the global total primary energy supply today^2^. Current research has largely reached a consensus that bioenergy deployment offers significant potential for climate change mitigation by reducing fossil carbon dioxide emissions^3,4^. According to the International Energy Agency, in the Net‐Zero Emissions by 2050 Scenario, modern bioenergy use will rise to 100 EJ in 2050, meeting almost 20% of total energy needs^5^.

Bioenergy, while the usage of which can mitigate climate change, may also be susceptible to future climate change. Therefore, the impact of climate change on bioenergy has been identified as a key area for further research^6–9^. Many of the studies to date, however, have focused on biomass potential^10–12^ or emission reduction potential^13–16^ while paying limited attention to such a critical field. Given that energy plants are one of the main sources of bioenergy, the climate-influenced changes in marginal land suitable for energy plants are analyzed to provide the basis for assessing bioenergy potential in the future^17^.

Thus far, only a few scholars have focused on the change in marginal land. Jiang et al.^18^ used multi-factor comprehensive analysis to investigate the spatial–temporal variation of marginal land suitable for energy plants from 1990 to 2010 in China. Jiang et al.^19^ assessed the marginal land availability for the Contiguous United States based on the land use change over the 2008–2015 period. The team focused mainly on past changes in marginal land due to the shifts in land use types. Aside from changing land use, climate change is another key factor affecting marginal land distribution as it alters the growing conditions of energy plants. Tuck et al.^20^ considered the effects of climate change and derived maps of the potential distribution of 26 promising bioenergy crops in Europe in the 1990s, the 2020s, 2050s, and 2080s. The above studies of change in marginal land used the multi-factor comprehensive analysis to set thresholds for different indicators according to crop growing conditions and extract the distribution of land resources that meets various growing requirements for crops, including climatic conditions, elevation, and so on^21^. Since the requirements of crop growing conditions often vary by region, the multi-factor comprehensive analysis can only obtain a rough distribution of marginal land at the macro level.

To obtain the accurate distribution of marginal land suitable for energy plants under current and future climate scenarios, more advanced methods should be employed. The machine learning methods that have proven to be effective in extracting land distribution were applied in this research, combining the occurrence records and growing conditions of energy plants to identify the global marginal land suitable for growing energy plants^22,23^. This in-depth analysis of the potential distribution of energy plants under the challenge of climate change proceeds in three steps: (1) choosing cassava, which is one of the most promising energy plants for bioenergy production, as a research object; (2) structuring the dataset with occurrence records and environmental covariates; (3) applying Boosted Regression Trees (BRT), a machine learning method, to evaluate the distribution of potential marginal land suitable for cassava from the perspective of environmental suitability under current and future climate scenarios. Our results contribute to a better understanding of potential marginal land resources of cassava across worldwide under different climate change scenarios, providing the basis for assessing bioenergy potential in the future.

## Materials and methods

In the present study, an ensemble boosted regression tree (BRT) modeling framework that has been used for mapping the environmental suitability of several bioenergy plants^23–27^ and medicinal plants^28,29^ was adopted. This modeling approach allows multicollinearity among covariates and can establish a multivariate empirical relationship between known occurrence points and the suitable environmental conditions in the corresponding locations where the target has been confirmed to have occurred. Further, this approach can map the environmental suitability of the target beyond the current geographical scope^30–33^. In this study, marginal land refers to land that has relatively poor natural conditions but is able to grow energy plants from environmental suitability and is not used for agricultural production. In order to estimate the implications of climate change for potential marginal land resources of worldwide cassava cultivation, the following datasets were used for modeling analysis in this study: (a) a comprehensive dataset of known cassava cultivation locations and a set of background samples representing locations where the environmental conditions are unsuitable for the growth of cassava; (b) a series of global environmental covariates considered to affect the growth of cassava. It is important to note that the WGS-84 geographical coordinate system was used, and all covariate layers were aggregated to a regular 0.05° × 0.05° global grid in this study.

### Data

#### Occurrence records and background samples

The known cassava cultivation locations (https://doi.org/10.15468/dl.y8f6fv) were acquired from the Global Biodiversity Information Facility (https://www.gbif.org) by combining the term “*Manihot esculenta* Crantz” with four filters (i.e., observation and occurrence). This comprehensive dataset includes 16,404 records of cassava occurrences with latitude and longitude information, which were used for modeling analysis. In the present study, these known locations of cassava cultivation were considered to reflect the suitable environmental conditions (i.e., climate, topography, and soil) for planting cassava. Based on the georeferenced information, these records were aggregated to 3845 grid units as the occurrence samples to match the spatial resolution of the related environmental covariates.

Both the occurrence and background samples are input as essential data to construct the BRT model^34,35^. Referring to the previously evaluated environmental suitability for cassava^27^, value < 0.5 was adopted as the standard to screen the background samples. Based on previous experience, the total quantity of background samples was set equal to the total number of occurrence samples. During the process, the randomly screening process was repeated 25 times through the seed function to reduce the impact of background samples on the estimation and to increase the robustness of the BRT models.

#### Environmental covariates

Several studies have revealed that environmental conditions play an important role in the growth of cassava^36,37^. In this study, three categories of environmental factors, including climate, topography, and soil, were adopted as the influencing factors of cassava environmental suitability.

*Bioclimatic factors*: The first category of environmental covariates contained 19 bioclimatic variables (i.e., annual mean temperature and annual precipitation). Given the evidence suggesting that both the metabolism of cassava and the life cycle of the bioenergy plant are susceptible to temperature and precipitation^38,39^, 19 bioclimatic variables were considered relating to the growth of cassava and were therefore selected as input datasets for the BRT models. All the bioclimatic variables of high spatial resolution across the globe spanning 1970 to 2000 are available on the WorldClim data website (https://www.worldclim.org/)^40^ In addition, the future bioclimatic variables at high spatial resolution generated from the MIROC-ESM-CHEM mode under three Representative Concentration Pathways (RCPs: RCP4.5, RCP6.0, and RCP8.5) can be acquired from the website of the Research Program on Climate Change, Agriculture and Food Security (https://ccafs-climate.org/data_spatial_downscaling/)^41^.

*Topography factors*: The second category of environmental covariates included several topography factors (i.e., slope and elevation), which are potential constraints on the growth of main energy crops^42^. For example, cassava is not suitable for growth in steep places since soil and water are easily eroded in these zones. The global digital elevation dataset at 3 arc second spatial resolution provided by the NASA Shuttle Radar Topographic Mission is available on the website of the Consultative Group on International Agricultural Research Consortium for Spatial Information (https://srtm.csi.cgiar.org)^43^. In addition, the spatial analysis tool in ArcGIS 10.6 was adopted to generate a global land surface slope dataset based on the global digital elevation dataset.

*Soil factors*: The last category of environmental covariates was soil factors, which are important limitations affecting the growth of cassava^44^. In this study, considering the relatively stable properties of soil, soil class and effective soil depth were adopted, which are currently available on the World Soil Information website (https://www.isric.org/)^45^.

### Modeling analysis

In the present study, the R (v 3.3.3) statistical programming environment and the extension packages (i.e., “gbm” and “dismo”) were employed to perform modeling procedures. The main parameters of BRT models were set referring to previous experience, the detailed description of which can be found in the study conducted by Jiang et al.^27^. Meanwhile, the ten-fold cross-validation method was used to avoid over-fitting and to select the optimal number of regression trees for modeling analysis. Based on the occurrence samples and background samples, 25 sub-models were constructed and further integrated into an ensemble BRT model. In addition, the receiver operating characteristic curve (ROC-AUC) and the relative contribution (RC) were adopted to evaluate the accuracy of the models and the importance of environmental covariates, respectively. It is also important to note that the current and future environmental suitability maps (with environmental suitability level ranging from 0 to 1) of cassava were generated using the ensemble BRT models and the corresponding bioclimatic data. All data management and analysis were conducted in R version 4.0.3. For visualization, maps were generated in ArcGIS version 10.6.

## Results

### Accuracy validation

**Figure 1:**
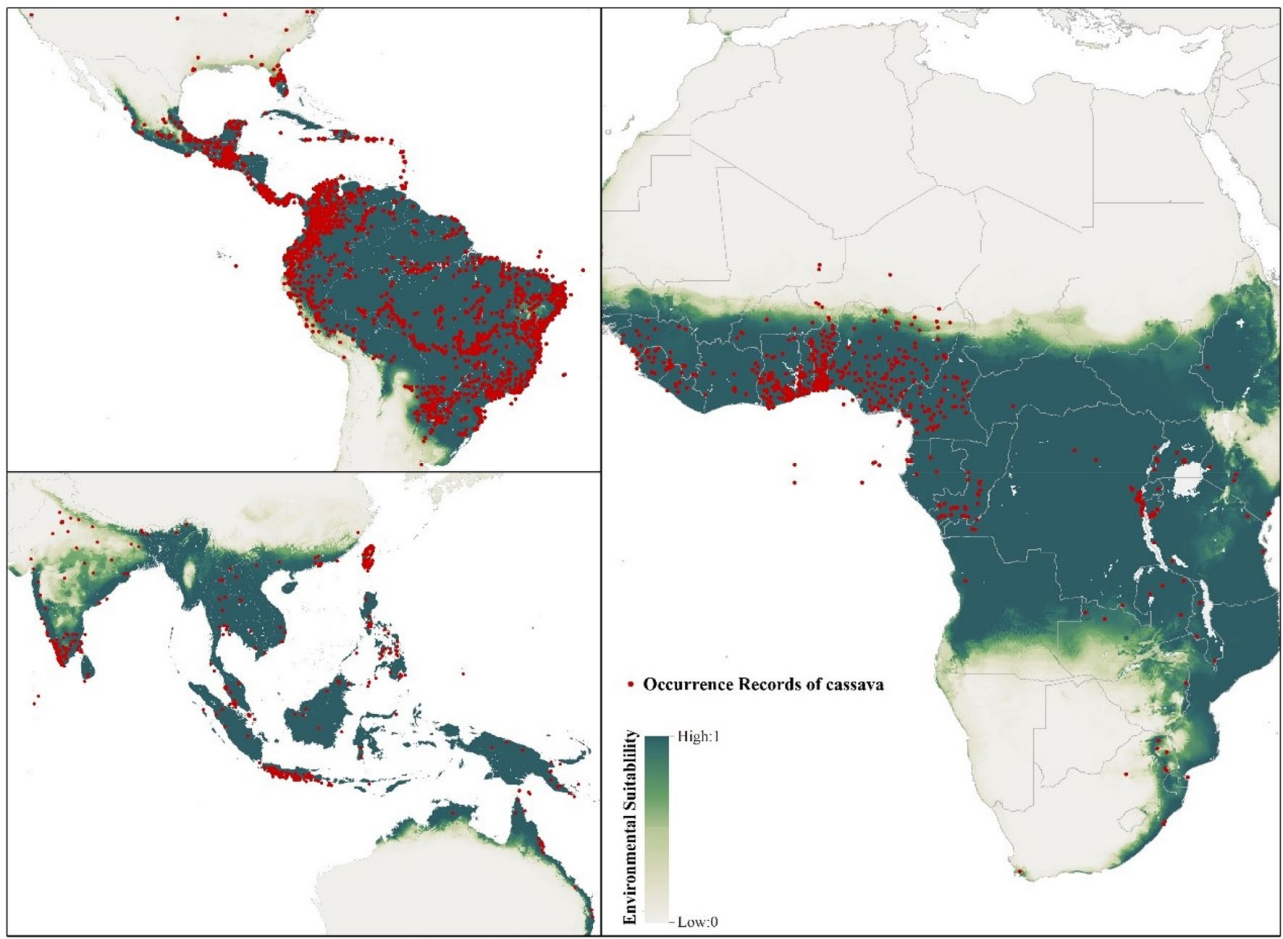
Distribution of the global cassava occurrence records matching the simulated global environmental suitability for cassava.

The worldwide environmental suitability for growing cassava was simulated using the BRT model constructed with 3845 records of cassava occurrence across the world. As can be seen in Fig. 1, these occurrence points were found mostly falling in regions predicted to be highly suitable for cassava cultivation, suggesting a reliable performance of our model. In addition, the value of AUC obtained from the tenfold cross-validation of 0.998 ± 0.001 could further indicate the high accuracy of our model simulation.

Moreover, the uncertainty of our spatial prediction was quantified by calculating the standard deviation values; the visualized map (Fig. S1) presented a low uncertainty, supporting the validity of the prediction.

### Relative contributions of environmental variables

Table 1 presents the relative contributions of the environmental variables that are larger than 1% to simulating the global environmental suitability for cassava cultivation. The results identify a substantial influence of temperature-related factors on evaluating the suitability, adding up to more than 90% of the total contribution. It is noteworthy that 76.06% ([95%CI 74.56–77.55%]) of the temperature-related contribution was from the temperature annual range, indicating its crucial role in the simulation process, followed by temperature seasonality (SD × 100) (9.82%, [95%CI 8.57–11.07%], mean temperature of coldest quarter (2.54%, [95%CI 2.20–2.87%]), and min temperature of coldest month (1.96%, [95%CI 1.65–2.28%]). On the other hand, precipitation seems to be of much less importance for suitability evaluation, the top two of which are precipitation of wettest quarter (4.14%, [95%CI 3.70–4.58%]) and annual precipitation (3.33%, [95%CI 3.05–3.61%]). In addition, the effects of soil and topographical factors are almost negligible, which collectively account for only 0.15% of the total influence on suitability prediction.

**Table 1:** Relative importance of non-climate factors and climatic predictors with > 1% contribution.

Environmental conditions	Environmental variables	Mean (%)	95%CI
Temperature-related	Temperature annual range	76.06	74.56–77.55
	Temperature seasonality (SD × 100)	9.82	8.57–11.07
	Mean Temperature of coldest quarter	2.54	2.20–2.87
	Min temperature of coldest month	1.96	1.65–2.28
Precipitation-related	Precipitation of wettest quarter	4.14	3.70–4.58
	Annual precipitation	3.33	3.05–3.61
Topography	Elevation	0.11	0.09–0.13
	Slope	0.03	0.03–0.04
Soil	Soil depth	0.01	0.00–0.01

**Figure 2:**
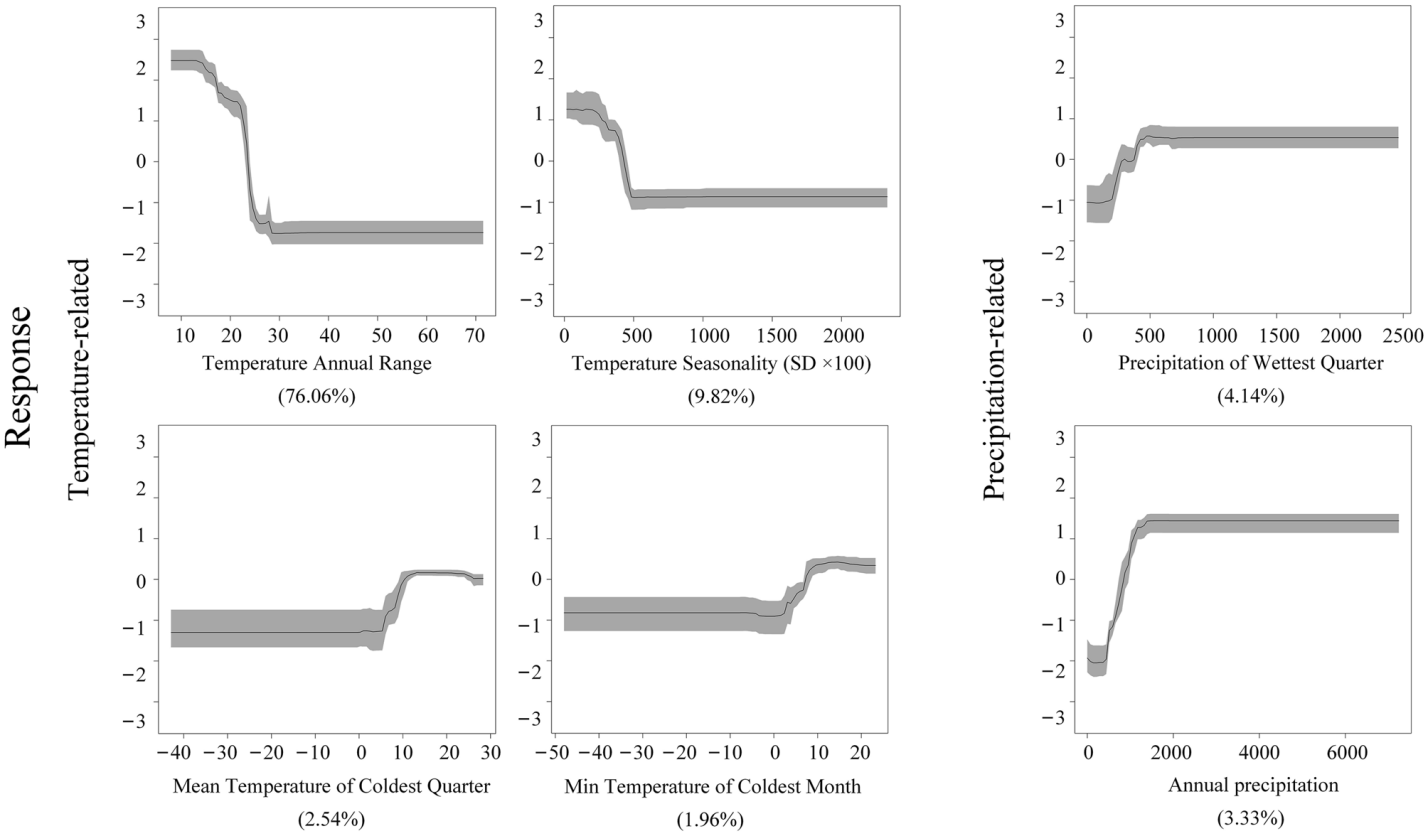
Marginal effect curves of major predicting variables in the ensemble BRT models for simulating environmental suitability of cassava planting.

The marginal effects of the major predictors are depicted in Fig. 2. Regarding the temperature-related variables, the environmental suitability for planting cassava is observed to be negatively associated with the temperature annual range before the number reaches 30 °C. Similarly, a negative relationship was also found between the environmental suitability and temperature seasonality before the value of its standard deviation hits 500 °C. On the other hand, a positive relationship was observed between the possibility of suitable land for cassava and the following two factors: mean temperature of the coldest quarter and the minimum temperature of the coldest month when the values are above 5 °C. Meanwhile, the two precipitation-related variables exhibit a positive association with the suitability for cassava cultivation until a certain point when precipitation of the wettest quarter rises above 500 mm or annual precipitation exceeds 1500 mm.

### The present potential marginal land resources for cassava

**Figure 3:**
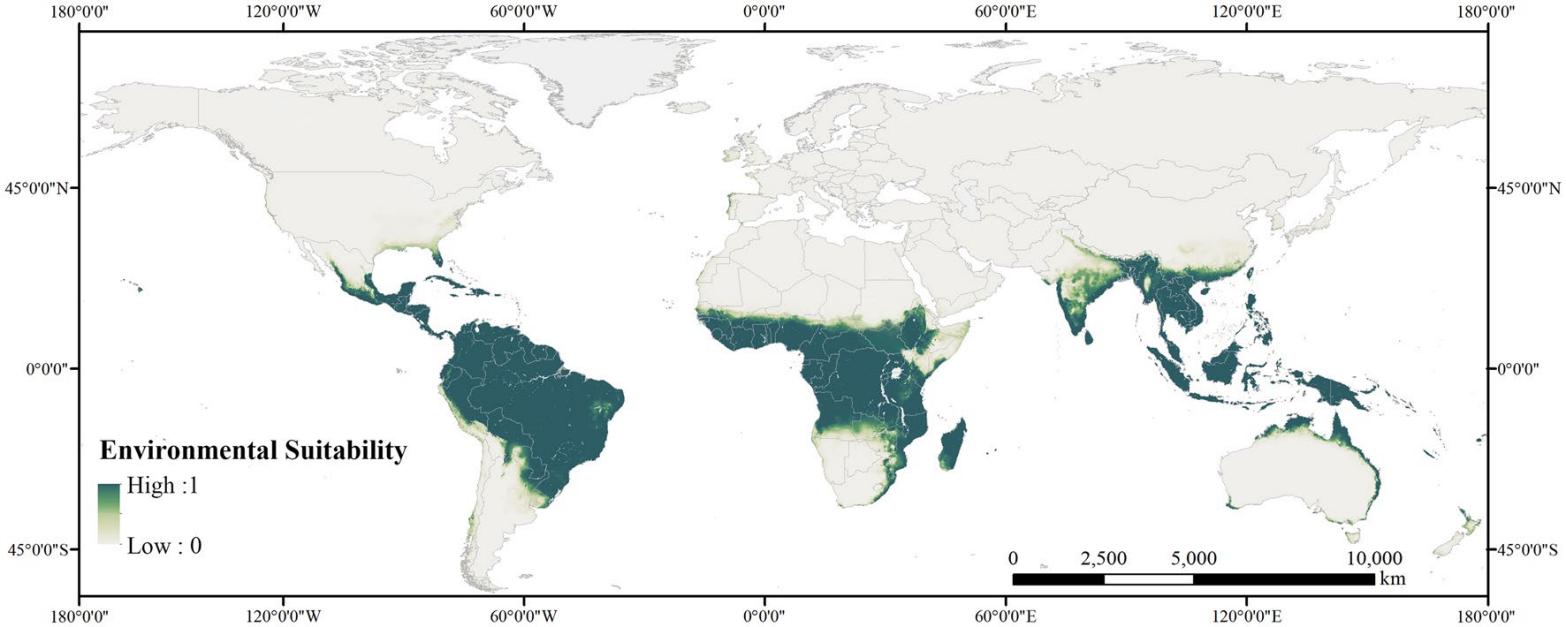
Simulated global environmental suitability for cassava cultivation.

The worldwide environmental suitability for cassava was visualized on the map using a dark green-light gray gradient that represents high to low (1–0) suitability (Fig. 3). The map shows that suitable areas are mainly distributed in tropical continents, ranging from inland to coastal regions, spanning a large part of South America, Africa, and the coasts of South and East Asia, and Oceania.

**Figure 4:**
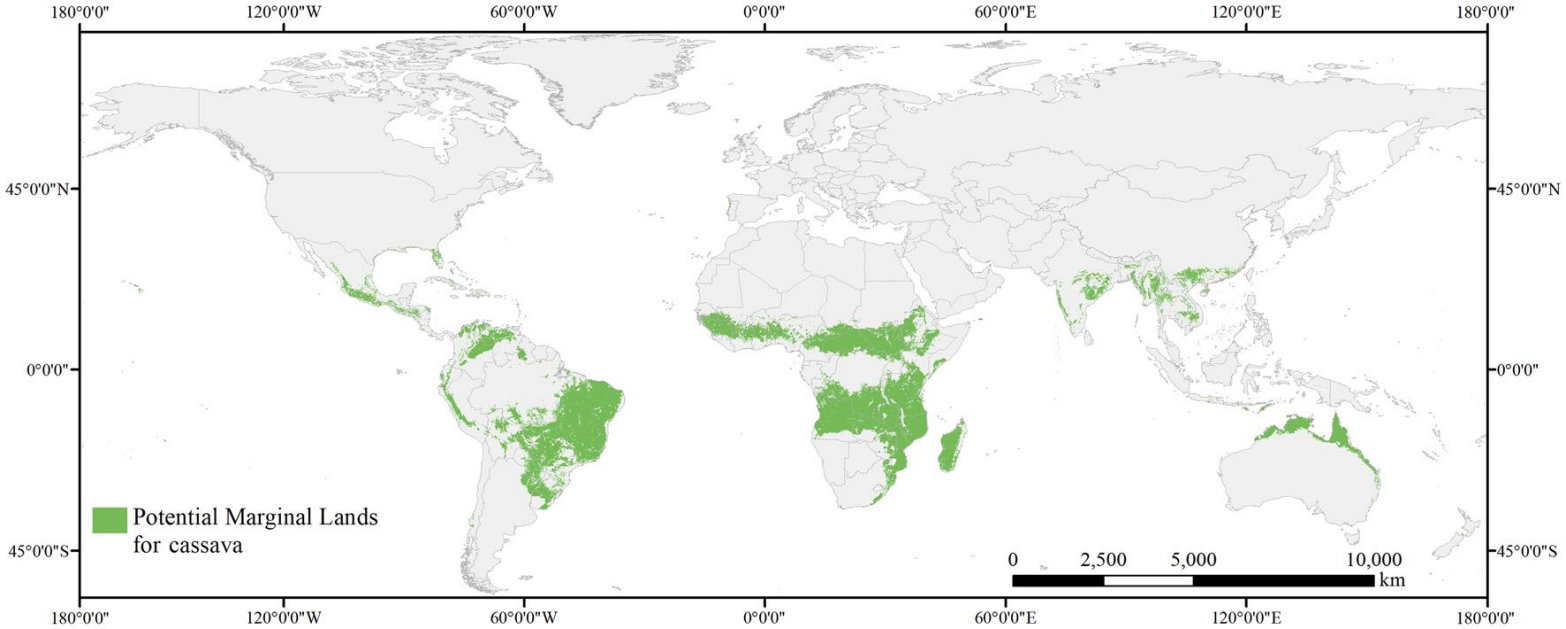
Global map of marginal land resources available for cassava cultivation.

To determine suitable land resources for planting cassava, the global suitability map was divided into 5 × 5 km^2^ grid cells, where only the grids that satisfy growing cassava can be distinguished by defining the threshold value of grids at 0.5. On this basis, land of productive or ecological value was subtracted when identifying available land resources for cassava cultivation to avoid conflicting agricultural or industrial production, leaving only marginal lands that include grasslands, savannas, and shrublands, as presented in Fig. 4.

By calculation, there are currently 1357.24 million hectares (Mha) of available marginal land for growing cassava, more than 80% of which are distributed in Africa and South America. The results of regions possessing top amounts of plantable marginal land resources for cassava are listed in Table 2 in descending order. As shown in this table, Africa owns 51.63%, the largest share of the total worldwide marginal land (700.70 Mha) for growing cassava, most of which is found in countries in or around Central Africa, such as the Democratic Republic of the Congo (79.95 Mha), Angola (79.22 Mha), Tanzania (66.61 Mha), and Mozambique (61.86 Mha). In addition, a considerably large proportion of land resources was observed in South America (464.60 Mha) that takes up 34.23% of the global marginal land available for cassava, more than 70% of which belongs to Brazil (332.75 Mha). Other than the land resources found in Africa and South America, there is also a small fraction of marginal land resources scattered in the rest continents. In Asia, such land resources are mostly distributed in countries in South and East Asia, consisting of marginal land in India (31.52 Mha), China (19.20 Mha), and Myanmar (15.81 Mha). In Oceania and North America, these land resources were mainly concentrated in a single country, which was Australia (63.42 Mha) and Mexico (22.51 Mha).

**Table 2:** Potential marginal land resources for cassava cultivation in major global regions and countries with more than 50 Mha of suitable marginal land.

	Region	Estimated potential area (Mha)	Percentage (%)
Continent	Africa	700.70	51.63
	South America	464.60	34.23
	Asia	90.07	6.64
	Oceania	64.28	4.74
	North America	36.68	2.70
	Europe	0.92	0.07
	Brazil	332.75	24.52
	Democratic Republic of the Congo	79.95	5.89
Country	Angola	79.22	5.84
	Tanzania	66.61	4.91
	Australia	63.42	4.67
	Mozambique	61.86	4.56

### Simulation of global environmental suitability for cassava in various climate scenarios

**Figure 5:**
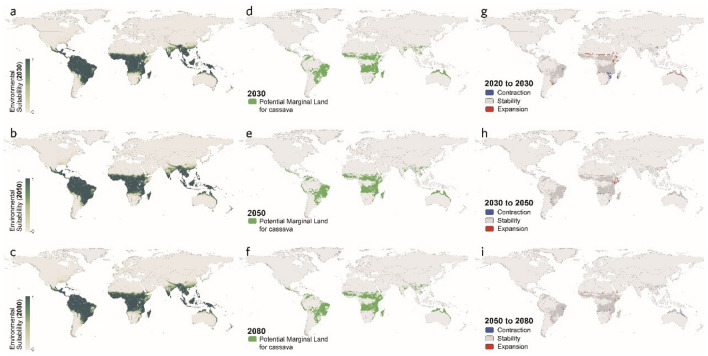
Global distribution of marginal land resources suitable for cassava cultivation in RCP4.5 scenario [2030 (**a**), 2050 (**b**), and 2080 (**c**)]. Variation in suitable marginal land for growing cassava (**d**–**f**).

**Figure 6:**
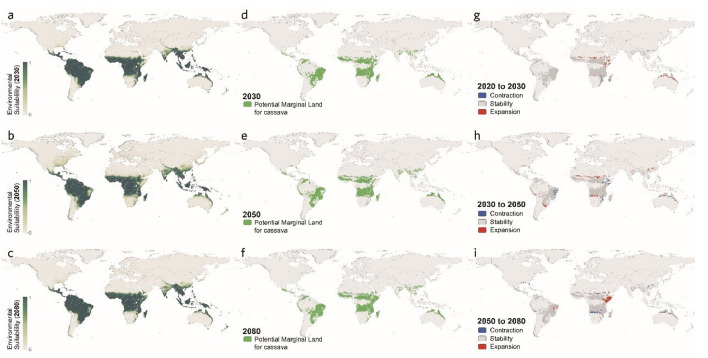
Global distribution of marginal land resources suitable for cassava cultivation in RCP6.0 scenario [2030 (**a**), 2050 (**b**), and 2080 (**c**)]. Variation in suitable marginal land for growing cassava (**d**–**f**).

**Figure 7:**
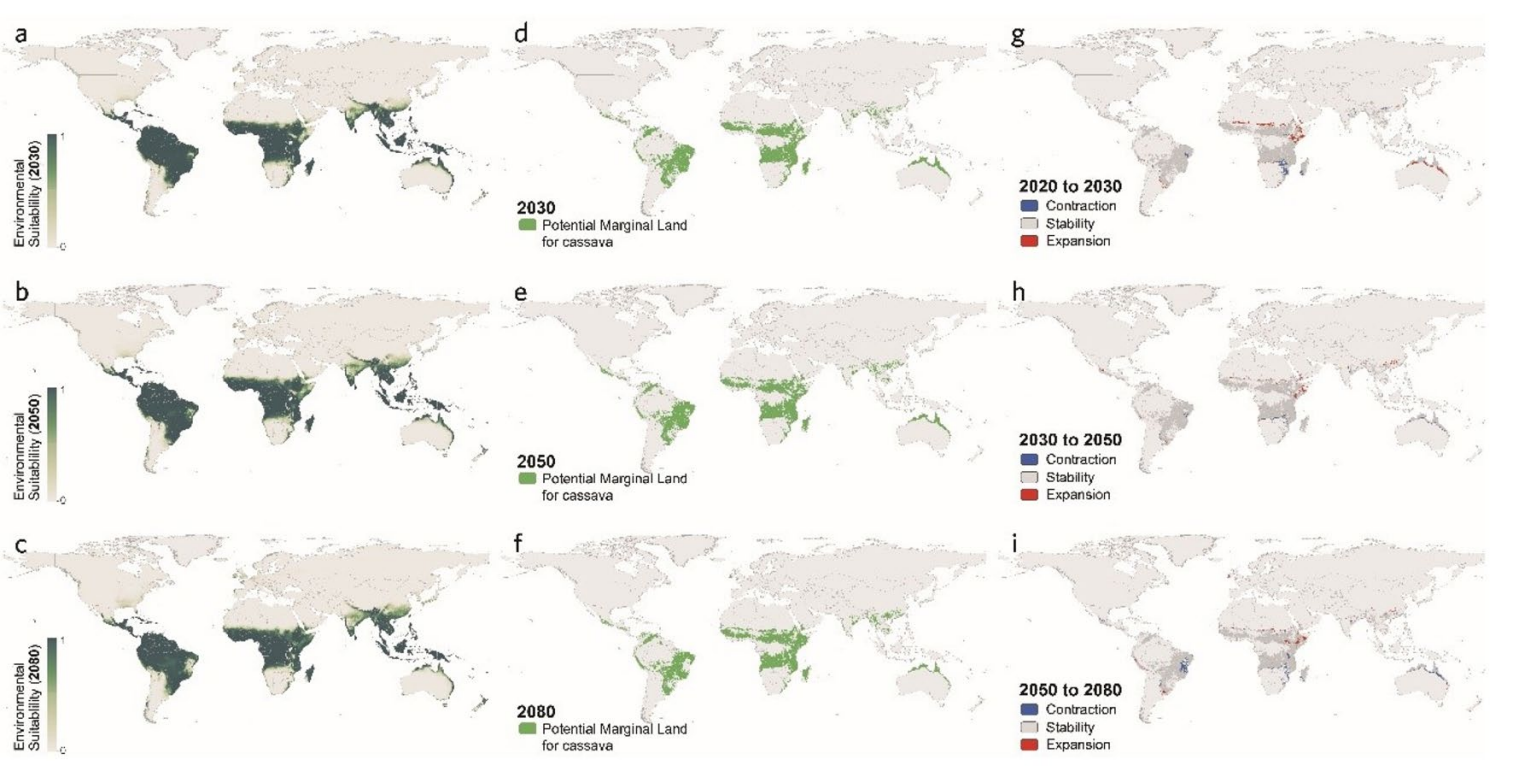
Global distribution of marginal land resources suitable for cassava cultivation in RCP8.5 scenario [2030 (**a**), 2050 (**b**), and 2080 (**c**)]. Variation in suitable marginal land for growing cassava (**d**–**f**).

The maps regarding all three climate scenarios exhibit relatively little variation in the global suitability for cassava cultivation. As shown in Figs. 5, 6, and 7, the highly suitable regions remain largely consistent with the global suitability distribution at present, with some fluctuations in certain areas, including the northeast corner of South America, the central, east, and south parts of Africa, South Asia, and the south part of East Asia, as well as the north coastal regions of Australia in Oceania.

In Table 3, through quantifying the global marginal land suitable for cassava by different climate scenarios, it is found that in the long term, such land resources would, statistically, experience the largest increase in the RCP6.0 scenario, followed by that in the RCP4.5 and RCP8.5 scenarios, which could be explained by a hypothetically more favorable growing conditions provided in the RCP6.0 scenario, given the heavy dependence of cassava growth on suitable temperature conditions.

**Table 3:** Potential marginal land resources for cassava by continent in different scenarios.

Continent	2030		2050		2080	
	Area (Mha)	Percentage (%)	Area (Mha)	Percentage (%)	Area (Mha)	Percentage (%)
RCP4.5 Scenario						
Africa	722.74	51.74	726.60	52.04	747.76	53.49
Asia	91.07	6.52	94.81	6.79	97.56	6.98
North America	33.30	2.38	33.50	2.40	32.51	2.33
Oceania	85.46	6.12	83.95	6.01	70.50	5.04
South America	463.59	33.19	456.59	32.70	448.09	32.05
Europe	0.80	0.06	0.78	0.06	1.64	0.12
Total	1396.95		1396.22		1398.06	
RCP6.0 Scenario						
Africa	749.32	52.64	750.27	52.09	776.31	53.28
Asia	92.02	6.46	104.27	7.24	101.52	6.97
North America	30.55	2.15	46.63	3.24	30.39	2.09
Oceania	92.26	6.48	90.55	6.29	85.43	5.86
South America	458.46	32.21	448.37	31.13	461.95	31.71
Europe	0.86	0.06	0.18	0.01	1.38	0.09
Total	1423.45		1440.26		1456.98	
RCP8.5 Scenario						
Africa	730.02	52.27	743.84	52.65	742.84	53.86
Asia	90.34	6.47	101.07	7.15	108.58	7.87
North America	29.80	2.13	31.09	2.20	27.80	2.02
Oceania	85.78	6.14	76.75	5.43	62.08	4.50
South America	460.02	32.94	458.39	32.45	433.00	31.40
Europe	0.60	0.04	1.55	0.11	4.83	0.35
Total	1396.55		1412.67		1379.11	

As shown in Table 3, in the RCP4.5 scenario, the worldwide suitable marginal land resources were predicted to grow by 39.71 Mha between now and 2030, remaining relatively stable in the 2030–2050 and 2050–2080 periods, with an overall 40.82 Mha-increase in the marginal land resources by 2080. In the RCP6.0 scenario, the global marginal land resources were estimated to rise by 66.21 Mha by 2030 and continue increasing in the next two periods, eventually growing by 99.74 Mha by 2080 compared with the current amount of 1357.24 Mha. In the RCP8.5 scenario, the growth of global land resources for cassava in the first two periods fails to be sustained in the last one, and by 2080, the increases in available land resources amount to 21.87 Mha from now.

**Figure 8:**
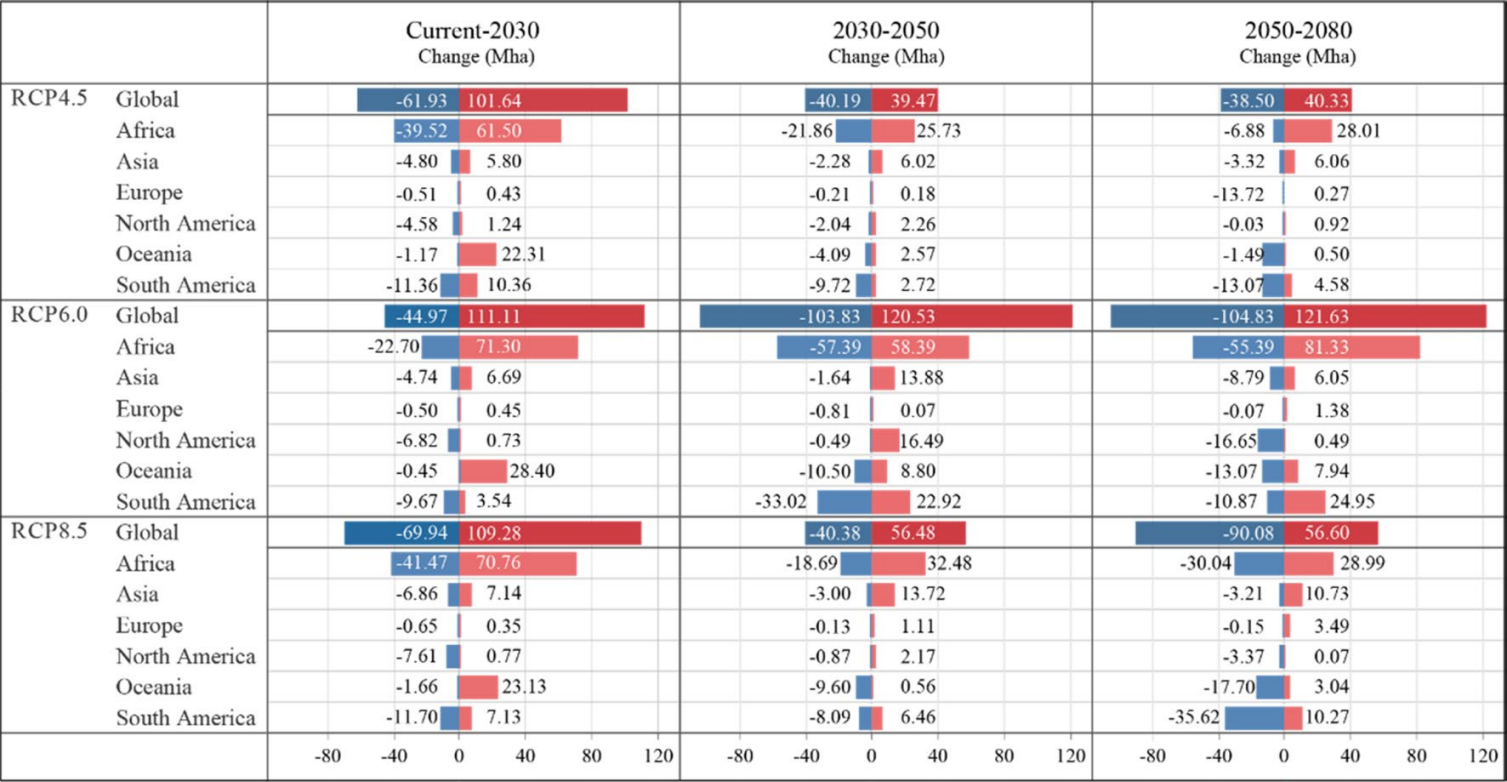
Potential marginal land resources for cassava by continent in different scenarios.

In addition, it is worth noticing that such statistics of the global marginal lands for growing cassava are the results of the expansion and the contraction of suitable areas canceling each other out, which were broken down specifically in Fig. 8 on scales from regional to global, exhibiting the greatest variation in RCP6.0 of both the expansion and the contraction, followed by that in RCP8.5 and RCP4.5. Moreover, by observing Fig. 8 and the above Figs. 5, 6, and 7, it is found that the substantial expansion would more likely take place in Central and East Africa, while the largest contraction tends to occur in Southern Africa and South America.

## Discussion

In this study, a machine learning method, BRT, was used to identify the potential marginal land suitable for cassava under current and various future climate change scenarios. In this research, 1357.24 Mha of marginal land was found currently available for growing cassava, which is generally consistent with the result of Jiang et al.^27^, suggesting that the area of marginal land suitable for cassava is approximately 1696.28 Mha. Besides, the overall distribution of suitable land resources is also relatively consistent with previous studies. According to the calculation of Fu et al.^46^ through multiple factor analysis, there is 112 Mha of marginal land suitable for cassava in Asia, which was similar to the result of this study that identified 90.07 Mha of marginal land in Asia. Zooming in China, Fig. 4 shows that the marginal land suitable for cassava is mainly located in the southern region of China, which is also consistent with the results of Zhuang et al.^47^. In addition, the total global marginal land available for cassava cultivation by 2050 in this study is calculated to be around 1400 Mha, falling within the range of worldwide available marginal land for biomass production (1170 to 2180 Mha by 2050) quantified in Rahman et al.’s research^48^. All the comparisons above suggest the reliability of the results in this research. It should be noted that sample bias of the occurrence records and several unconsidered factors (i.e., protected areas) may add uncertainty to our results to some extent.

The results of relative contribution showed that temperature-related factors were the most important factors affecting the potential distribution of cassava, thusly indicating significant impacts of climate change on the potential distribution of energy plants. Therefore, it is necessary to consider the impacts of climate change on marginal land resources suitable for energy plants when assessing the future energy landscape for the purpose of achieving Net‐Zero Emissions in 2050. Further, a quite complex relationship was found between the potential distribution of marginal land suitable for energy plants and temperature-related factors. For example, the possibility of land being suitable for cassava was positively correlated with the mean temperature of the coldest quarter, yet negatively correlated with the temperature annual range before the value reaches 30 °C. As a result, the trends in marginal land change vary across different climate change scenarios and different countries or regions.

Although there is a general trend of growing marginal land in the future found in this research, the available marginal land is not guaranteed to increase. Marginal land use is such a complex issue that the environmental risks, ecosystem services, and sustainability of marginal land should be considered simultaneously^49^. For example, bioenergy production requires large amounts of water, which may increase the pressure on water resources, especially in countries that already suffer from water crises^50^. Besides, developing bioenergy may not be the most effective way in all regions to achieve energy savings and GHGs emission mitigation^51^. In addition, there could be a potential conflict between increasing food demand with population growth versus reduced yield under climate change with producing biomass for energy, especially for developing countries. In the future, marginal land should be exploited sustainably by giving full consideration to the actual situation of each region, including water supply and food security.

## Conclusion

In this study, the BRT model was used to evaluate the potential distribution of marginal land resources suitable for cassava under current and various future climate change scenarios. The results show that there is currently 1357.24 Mha of available marginal land for growing cassava, more than 80% of which is distributed in Africa and South America. The available marginal land resources for cassava are expected to remain within the range of 1396.55 to 1423.45 Mha in 2030, 1396.22 to 1440.26 Mha in 2050, and 1379.11 to 1456.98 Mha in 2080 under different climate scenarios. It should be noted that despite the general trend of increasing marginal land for bioenergy development in the future, several environmental and social constraints on exploiting marginal land resources need to be considered, such as water resources and food security.

### Supplementary Information

Supplementary Figure S1.

## Data Availability

All data used in this study is available from the corresponding author on reasonable request.
