# Supplementary material for: The implications for potential marginal land resources of cassava across worldwide under climate change challenges
Source: Sci Rep. 2023 Sep 13;13:15177. doi: 10.1038/s41598-023-42132-y (PMC10499798; doi:10.1038/s41598-023-42132-y)
Supplement: Supplementary file 1 — Supplementary Figure S1. [file 41598_2023_42132_MOESM1_ESM.docx]

# Supplementary Information


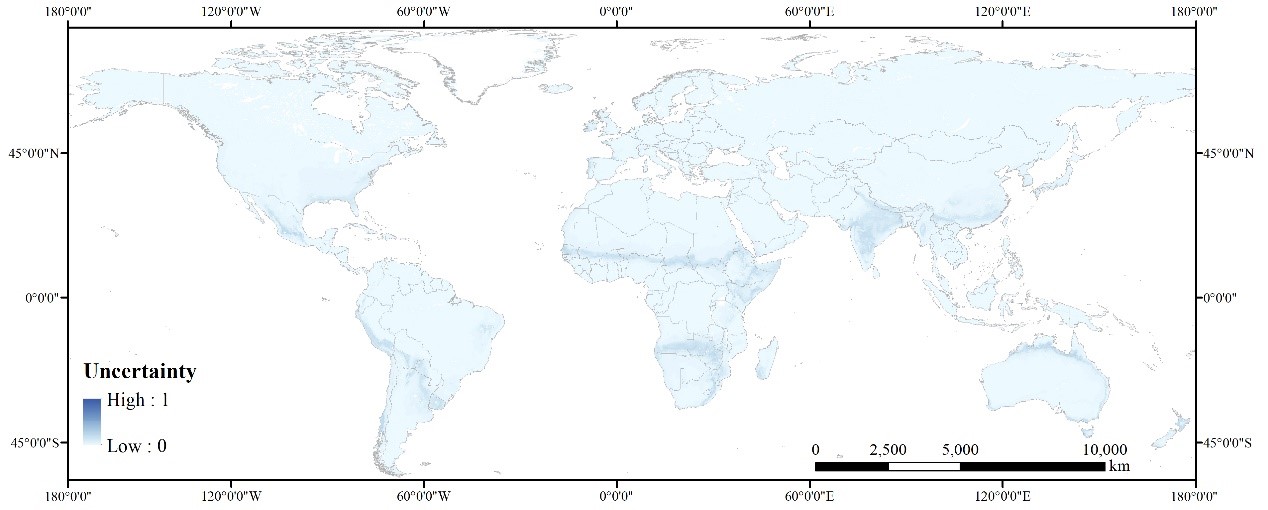


**Figure S1. Uncertainty in spatial prediction visualized from standard deviation values computed for each pixel across the model ensemble.**
